# New Updates Pertaining to Drug Delivery of Local Anesthetics in Particular Bupivacaine Using Lipid Nanoparticles

**DOI:** 10.1186/s11671-016-1520-8

**Published:** 2016-06-24

**Authors:** Siavash Beiranvand, Ali Eatemadi, Arash Karimi

**Affiliations:** Department of Anesthesiology, Lorestan University of Medical Sciences, Khoramabad, Iran; Department of Medical Biotechnology, School of Advanced Technologies in Medicine, Tehran University of Medical Sciences, Tehran, Iran

**Keywords:** Local anesthetics, Drug delivery, Lipid nanoparticles, Bupivacaine, Toxicity

## Abstract

Lipid nanoparticles (liposomes) were first described in 1965, and several work have led to development of important technical advances like triggered release liposomes and drug-loaded liposomes. These advances have led to numerous clinical trials in such diverse areas such as the delivery of anti-cancer, antifungal, and antibiotic drugs; the delivery of gene medicines; and most importantly the delivery of anesthesia drugs. Quite a number of liposomes are on the market, and many more are still in developmental stage. Lipid nanoparticles are the first nano-medicine delivery system to be advanced from laboratory concept to clinical application with high considerable clinical acceptance. Drug delivery systems for local anesthetics (LAs) have caught the interest of many researchers because there are many biomedical advantages connected to their application. There have been several formulation techniques to systemically deliver LA that include encapsulation in liposomes and complexation in cyclodextrins, nanoparticles, and to a little extent gold nanoparticles. The proposed formulations help to decrease the LA concentration utilized, increase its permeability, and most importantly increase the localization of the LA for a long period of time thereby leading to increase in the duration of the LA effect and finally to reduce any local and systemic toxicity. In this review, we will highlight on new updates pertaining to drug delivery of local anesthetics in particular bupivacaine using lipid nanoparticles.

## Review

### Introduction

A drug delivery system should have reduced tissue reaction, an efficient drug release capability, and a steady degradation rate for biodegradable carrier until all non-harmful products are passed out [1, 2]. For local anesthetics (LAs), the formulations of new potent delivery systems is aimed at modulating and controlling the release rate of these drugs, improve their anesthetic effect, and enhance their localization thereby reducing side effects associated with systemic toxicity [3]. LAs are important clinically for anesthesia and analgesia for management of acute and chronic pain after surgery and also reducing systemic toxicity and blocking sensory fibers though they may only last for few hours [4, 5].

For an excellent drug delivery system for LA to be designed/formulated, previous research studies have outlined two important factors that must be considered: (1) the drug has to be adequately transported in order to sustain a therapeutic concentration for a maximum period of time and (2) carrier/drug excretion must be minimally reduced for a decreased systemic concentration of the drug and its concentration on the site of injection [6]. It should also be noted that drug delivery systems for LA should act as a store house at the injection site in which the carried LA are slowly and gently released from these store house thereby reducing risk of increased plasma levels, increased period of nerve block, and decreased risk of systemic toxicity which is of great importance to patients with surgical or chronic pain [7, 8].

Recently, the application of local anesthetic drugs to provide postsurgical analgesia has been welcomed by great scientific and clinical interest [9]. However, local anesthetic drugs have short action period, so neural blockade enhancers like dexamethasone are often administered in combination with these anesthetic drugs. There are varieties of local anesthetics available including lidocaine, bupivacaine, and ropivacaine being some of the most commonly used [10]. Bupivacaine and ropivacaine have demonstrated to produce longer peripheral neural blockade (4.5–12 h) than lidocaine (1–2 h) as such bupivacaine is commonly used for long-acting anesthetic effects; however, it is also linked with compelling cardiotoxicity and neurotoxicity [11]. The toxicity of bupivacaine has been overcome by ropivacaine, the propyl analog of bupivacaine while still maintaining the same duration of action [12]. Additionally, ropivacaine shows a lower lipid solubility and vasodilation when compared with bupivacaine resulting in their increased circulation time within the delivered local environment [13]. While promising, local anesthetics need a continuous admixture to maintain and sustain an efficient postsurgical pain management, or else it can result to complex side effects like adverse local tissue reactions and systemic toxicity [14]. The application of biomaterials to develop a controlled release technique for local anesthetics has been efficient to deliver a non-lethal, localized, and postsurgical pain management system [15]. Over the years, researchers have formulated quite a number of biomaterial-based carriers, including liposomes microparticles and nanoparticles. These materials have been proven to be efficient in the release of encapsulated drugs and in addition can diffuse freely from the injection site [16].

### Types of Local Anesthetics

Local anesthetics (LAs) are used mostly for anesthesia and analgesia after surgery or for pain management, which normally last for a few hours. To prolong the duration of local anesthesia, the use of catheter techniques, disposable pumps, or multiple injections can be adopted [D]. Prolonged LA action has only resulted in doubled or tripled plain drug effect time, together with the use of adjuncts with LA agents of readily available agents.

**Fig. 1:**
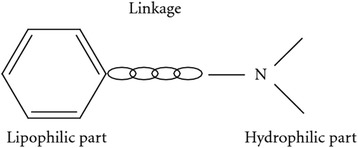
Basic structure of local anesthetics

**Fig. 2:**
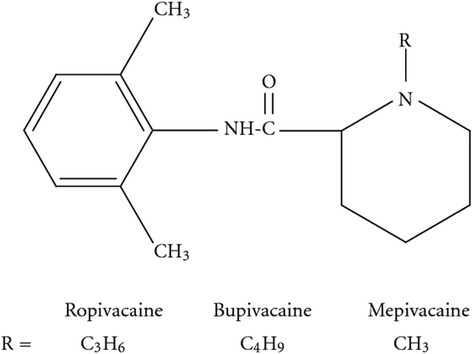
Amide local anesthetics

Local anesthetics can be categorized into two groups based on the nature of the chemical link, namely amides [−NH–CO–] and esters [−O–CO–] (Figs. 1 and 2). The amide group is more clinically important; it includes lignocaine, prilocaine, levobupivacaine, bupivacaine, mepivacaine, and ropivacaine.

**Fig. 3:**
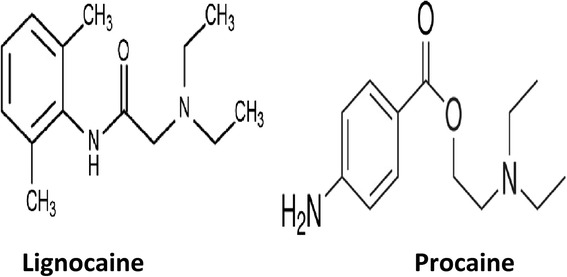
Amide local anesthetic (lignocaine) and ester local anesthetic (procaine)

The ester group of LA includes cocaine, procaine, chloroprocaine, and amethocaine, and they have been reported to be weak bases and solubilized for injection as strong conjugate acidic hydrochloride salts at a of pH 3–6 by (Fig. 3) [17, 18].

For topical and mucous formulations, benzocaine and butamben are the most commonly used ester type of local anesthetics. Dewachter reported the clinical advantage of amide group over ester group in that anaphylaxis to local anesthetics is very rare and has reduced the frequency of acute allergic reaction because of the decreasing use of the ester group of local anesthetics [19]. In addition, the metabolic product of the ester local anesthetic like para-aminobenzoic acid is mostly responsible for allergic reactions [19]. Cross-reactivity among esters is common.

However, there are allergic reactions associated with amide local anesthetics but remain anecdotal. It should be noted that ingredients like antioxidants or preservatives included in local anesthetic may also produce allergic or adverse reactions [19].

### Mechanisms of Action of Local Anesthetics

Local anesthetics directly block transmission of pain sensation from nociceptive afferent nerves. Local anesthetic agents are injected directly, and their potency results from action on the nerve where the inward sodium ion (Na+) current is blocked at the sodium ionophore during depolarization process [20]. LAs are also responsible for blocking calcium ions (Ca^2+^) and potassium ions (K^+^) channels and potential vanilloid-1 receptors [21]. Local anesthetics also alter the linking bond between some certain G proteins and their associated chemical receptors; so through this ability, LAs are able to carry out their anti-inflammatory effects, especially on neutrophil priming reactions [22].

### Lipid Nanoparticle/LA Drug Interaction

**Fig. 4:**
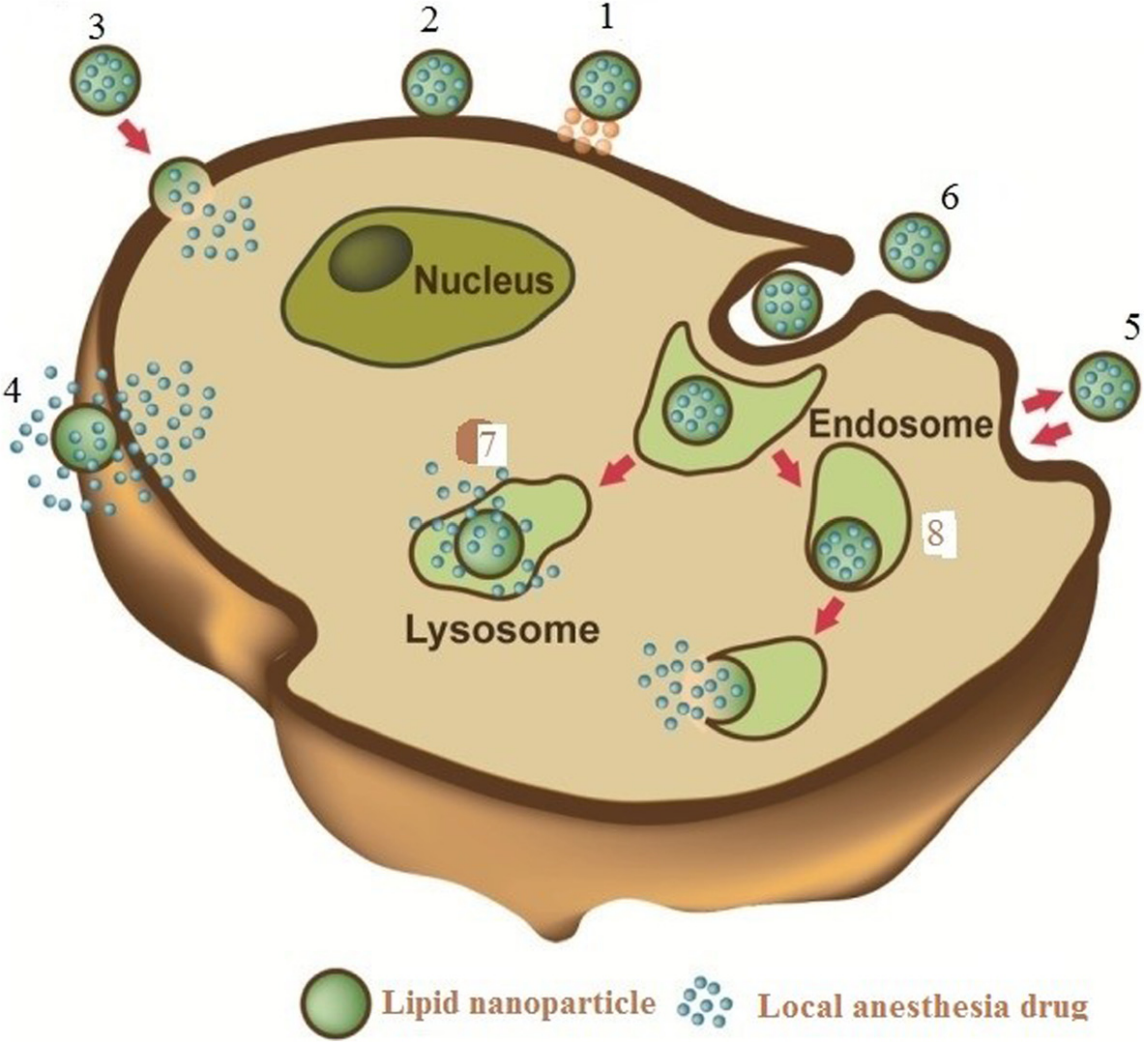
Lipid nanoparticle-cell interaction

When a lipid nanoparticle loaded with an anesthesia drug interacts with a cell, the delivery of the local anesthesia drug and its distribution in the target cell can be achieved in several ways: lipid nanoparticle loaded with an anesthesia drug interacts with the cell, binding to the surface via the receptors (1). Absorption onto the plasma membrane can also occur by electrostatic interactions (2). The delivery of the cargo into the cell cytoplasm can take place through different ways. Lipid nanoparticles fuse with the plasma membrane of the cell and empty the anesthesia drugs into the cell (3). After the interaction with the cell, the structure of the lipid nanoparticle bilayer can be affected and the anesthesia drug is released (4). Exchange of carrier-lipid components with the cell membrane can also occur (5). Lipid nanoparticles engulfed by endocytosis (6) can either follow these paths: endosomes fuse with lysosomes (7): in this case, the anesthesia drug is released as a result of breakdown of the liposome membrane by low PH. Endosomes follow another route (8): lipid nanoparticle releases their loaded drug after the degradation of the endocytic vesicle (Fig. 4) [23].

### Toxicity of Local Anesthetics

Systemic toxicity is caused by increased concentrations of local anesthetics. They happen when there is an accidental discharge of anesthesia drugs into the systemic circulation. The central nervous system is the most prone and sensitive to the toxic action of local anesthetic agents and cardiovascular system and can result into symptoms like isolated muscle contractures, incoherent speech, generalized convulsions, unconsciousness, etc. [24]. Recently, Cai and his colleagues demonstrated in their study that bupivacaine, tetracaine, and etidocaine increases tendency and possibilities to impair the proper functioning of both the cardiovascular system and central nervous system compared with lidocaine, mepivacaine, and prilocaine [25]. The central nervous system is more sensitive and prone to the toxic effects of local anesthetics than the cardiac system because it depends upon sodium channels for proper functioning, and it is the first to typically manifest signs of toxicity above the normal pharmacological dose of the administered LA drug (Table 1).

**Table 1:** Showing the pharmacological data for the two LA groups

LA groups	Max dose (mg/kg)	Duration (h)
Esters		
Chloroprocaine	12	0.5–1
Procaine	12	0.5–1
Cocaine	3	0.5–1
Tetracaine	3	1.5–6
Amides		
Lidocaine	4.5/(7 with epi)	0.75–1.5
Mepivacaine	4.5/(7 with epi)	1–2
Prilocaine	8	0.5–1
Bupivacaine	3	1.5–8
Ropivacaine	3	1.5–8

### Toxic Effects on Central Nervous System

The initial CNS symptoms are tinnitus, blurred vision, dizziness, tongue parathesias, and circumoral numbness. Excitatory signs such as nervousness, agitation, restlessness, and muscle twitching are the result of blockade of inhibitory pathways. While the early signs advance to CNS depression with slurred speech, drowsiness, unconsciousness, and then respiratory arrest.

### Toxic Effects on Cardiovascular System

Local anesthetics have directed toxic effects on the heart and peripheral blood vessels. They block the fast sodium channels in the fast-conducting tissue of Purkinje fibers and ventricles leading to a reduced rate of depolarization as well as the effective refractory period and action potential duration (https://www.openanesthesia.org/local_anesthetics_systemic_toxicity/).

### Liposomal Bupivacaine and Its Functional Platform for Anesthesia Drug Delivery

Recently, liposomal bupivacaine has been applied in clinical practice and has been investigated in many clinical trial studies on healthy patient volunteers in order to procure a long-lasting pain relief in a single dose administration (DepoFoam bupivacaine, Exparel™) (Table 2). Liposomal bupivacaine is made up of liposomal spheres with a radius size of 15.6 μm ± 17.8 [26].

**Table 2:** Summary of liposomal bupivacaine application

Author	Study design	Result
McAlvin et al. [51]	To evaluate the effect of liposomal bupivacaine on the sciatic nerve in experimental models	Histological evaluation reported that both Exparel complex and bupivacaine hydrochloride produce tissue reaction with the liposomal complex being less aggressive.
Lonner et al. [52]	To study the role of liposomal bupivacaine in pain management after total joint arthroplasty and observed that liposomal bupivacaine pharmacokinetics and pharmacodynamics	Liposomal bupivacaine has less cardiac toxic effects, less cardiac toxic effects, without significant differences between Exparel™ and placebo: palpations and extrasystoles (≤2 %), tachycardia (3.9 %), bradycardia (1.6 %), hypertension and hypotension (≤2 %).
Richard et al. [53]	To study any hematological, biochemical, and biological side effects of Exparel complex in laboratory animals	(1) Histological analysis reported that there was evidence of granulomatous inflammation, 15 days after administration of Exparel™ formulation. (2) There was maintenance of optimum plasma concentration for about 96 h. (3) Administration of liposomal bupivacaine to postoperative patients reduces the intake of opioids, the hospital admission period, and costs of bills.
Soberón et al. [49]	Study was carried out on a 45-year-old woman with digital ischemia on the ring finger and little finger at the right hand.	(1) It was reported that the results were superior to the subclavicular block due to a better and efficient pain control. (2) It was reported that after surgery, photo-plethysmography, showed a normal ulnar artery and loss of finger cyanosis, as a result of vasodilation effect produced by liposomal bupivacaine.

Recent in vitro studies comparing the Exparel™ complex with bupivacaine hydrochloride 0.5 % (*w*/*v*) and 1.31 %, respectively, regarding controlled release system, with the same amount of active substance have been carried out, and it was reported that the release kinetics profile was different in the case of hydrochloride from free bupivacaine with a peak time release of 48 h, while concerning Exparel™ complex and the peak time release was about 800 h [27] (Table 3).

**Table 3:** Examples of marketed liposomal for anesthesia drug and other drugs

Trade name	Nanoparticle platform	Drug	Size	Indication
Anti-cancer				
Doxil/Caelyx (Janssen)	PEG-liposomes	Doxorubicin	100 nm	Kaposi’s sarcoma, ovarian cancer, breast cancer, combination with bortezomib in multiple myeloma
DaunoXome (Galen)	Lipid nanoparticle	Daunorubicin	45–80 nm	Kaposi’s sarcoma
DepoCyt (Pacira)	Lipid nanoparticle	Cytarabine	20 μm	Malignant lymphomatous meningitis
Marqibo (Talon)	Lipid nanoparticle	Vincristine	100 nm	Acute lymphoblastic leukemia
Myocet (Cephalon)	Lipid nanoparticle	Doxorubicin	80–90 nm	Combination therapy with cyclophosphamide in breast cancer
Analgesics				
Diprivan (Fresenius Kabi)	Lipid emulsion	Propofol	180 nm	Anesthesia
DepoDur (Pacira)	Lipid nanoparticle	Morphine	17–23 μm	Postsurgical pain
Exparel (Pacira)	Lipid nanoparticle	Bupivacaine	24–31 μm	Anesthesia
Antifungal				
Abelcet (Sigma-Tau)	Lipid drug particles	Amphotericin B	2–5 μm	Aspergillosis
AmBisome (Astellas)	Liposome	Amphotericin B	<100 nm	Antifungal, leishmaniasis

**Fig. 5:**
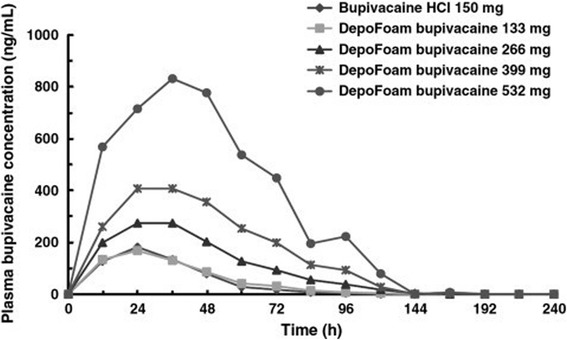
Plasma bupivacaine concentration after administration of DepoFoam bupivacaine or bupivacaine HCl to patients undergoing total knee arthroplasty

In Bramlett et al.’s study, bupivacaine-loaded lipid nanoparticle was demonstrated to provide benefit in total knee arthroplasty (TKA); in that study of 138 TKA, Bramlett compared several doses of DepoFoam (lipid nanoparticle bupivacaine) against bupivacaine hydrochloric acid (HCl) in wound infiltration [28]; it was reported that the highest dose of bupivacaine-loaded lipid nanoparticle of 532 mg was the only effective dose that was capable of producing improved pain scores in the TKA patients at rest over the bupivacaine, and was found to be dose-related [29] (Fig. 5) (Table 4).

**Table 4:** Summary of studies assessing the efficacy of bupivacaine-loaded lipid nanoparticle

Author	Study design	Surgery	Intervention/Placebo	Primary end point	Results
Gorfine et al. [54]	Random controlled trial	Hemorrhoidectomy	DepoFoam Bupivacaine 300 mg compared with 0.9 % sodium chloride	Numerical rating score (NRS), AUC 0–72 h	Pain intensity scores were significantly decreased in the extended release bupivacaine group versus placebo (141.8 vs. 202.5, *P* < 0.001).
Smoot et al. [55]	Random controlled trial	Mammoplasty	DepoFoam bupivacaine 600 mg compared with bupivacaine HCL 200 mg þ epinephrine 1:200,0000	NRS-Activity, AUC 0–72 h	No statistical difference between the groups (AUC NRS-A, 441 vs. 468, P 1⁄4 0.3999).
Golf et al. [29]	Random controlled trial	Bunionectomy	DepoFoam 120 mg compared to 0.9 % sodium chloride	NRS AUC 0–24 h	Pain intensity score was significantly decreased in lipid nanoparticle bupivacaine versus control, 123.9 in DepoFoam versus 146 in placebo, *P* < 0.0005.
Bramlett et al. [28]	Random controlled trial	Total knee arthroplasty	DepoFoam bupivacaine (133, 266, 399, and 532 mg) compared to bupivacaine HCl 150 mg with epinephrine 1:200,000	NRS-A, AUC 0–96 h	No statically significant difference between all DepoFoam groups versus bupivacaine HCl (*P* > 0.05).
Cohen et al. [56]	Cohort study	Colectomy	DepoFoam 366 mg compared with postoperative PCA	Total milligrams of opioids consumed after surgery and total cost of hospitalization	Mean total amount of postsurgical opioids significantly less in DepoFoam compared to PCA group (57 vs. 115 mg, P 1⁄4 0.025). Average cost of hospitalization significantly less in DepoFoam versus PCA (US$ 8766 vs. US$ 11,850 P 1⁄4 0.027).

**Fig. 6:**
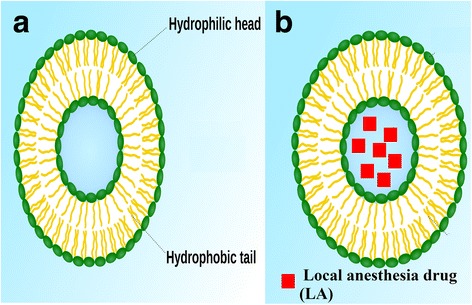
Lipid nanoparticle (**a**) and a loaded lipid nanoparticle with local anesthesia drug (**b**)

Lipid nanoparticles have several characteristics such as biodegradable, decreased toxic, and solubilized drug delivery system that have been widely utilized in biomedical field, also lipid nanoparticle formulations have demonstrated several abilities to improve on the pharmacokinetics and pharmacodynamics of the loaded encapsulated drugs; in addition, it have been showed to be a potent drug carrier for several drugs like insulin, sildenafil citrate, amphotericin B, and methotrexate [30] (Fig. 6). Presently, there are few or no research studies that have investigated the encapsulation of LA into lipids nanoparticles [31]. However, there are few available clinical studies that have investigated and compared the differences between EMLA (lidocaine 2.5 % and prilocaine 2.5 %) and liposomal systems for several local anesthesia agents (Table 4). Recently, lipid nanoparticle bupivacaine has been investigated to provide up to 72 h of analgesia effect after hemorrhoidectomy surgery, thereby decreasing administration of opioids.

One of such research studies results reported by Bucalo and his colleagues stated that liposomal lidocaine preparations is more efficient than non-lipid nanoparticle vehicles in terms of increased time duration of anesthesia [32]. Furthermore, other studies have also investigated clinical comparisons between EMLA (lidocaine 2.5 % and prilocaine 2.5 %) and 5 % lipid nanoparticle tetracaine for skin anesthesia. Taddio and his research teammates demonstrated the effect of local anesthesia of a new lipid nanoparticle formulation made up of 4 % lidocaine in a randomized controlled trial, and it was reported that the lipid nanoparticles formulation exhibited a brief action duration of about 30 min, being potent for cutaneous analgesia in children [33]. Another research study on the use of lipid nanoparticle to encapsulate anesthesia drugs reported that after the injection of lipid nanoparticle bupivacaine, increased plasma concentrations were sustained for longer time period with the liposomal bupivacaine when compared with plain bupivacaine [34].

In another recent clinical study, a large multi-vesicular bupivacaine formulation was investigated in six healthy volunteers through intradermal injections in various injection sites like the lower back, with different percentages of 0.5, 1.0, and 2 % of lipid nanoparticle bupivacaine; 0.5 % plain bupivacaine; 0.9 % saline; and sham liposomes. The calculated median time duration of analgesia effect with 0.5 % bupivacaine was 1 h; with 0.5, 1.0, and 2.0 % liposomal bupivacaine; this was increased to 19, 38, and 48 h, respectively [35].

The introduction of lipid nanoparticle bupivacaine has paved a way for several randomized trials investigating the potency and safety of this new delivery system (Table 3). Gorfine et al. carried out a multicenter, randomized, double-blind, parallel-group placebo-controlled phase 3 research study to compare the potency and time duration of postsurgical analgesia effect from a single dose of lipid nanoparticle bupivacaine with placebo administered intra-operatively in patients undergoing hemorrhoidectomy. In this study, all the patients were randomly assigned to receive DepoFoam bupivacaine placebo made up of 30 ml of 0.9 % sodium chloride and 10 mg of morphine was administered intramuscularly as postsurgical pain control for every 4 to 6 h as necessary for the first 3 days of the postsurgical period [36, 37]. The primary end point and secondary outcome of this study are summarized in Table 4. Conclusively, it was reported that the administration of lipid nanoparticle bupivacaine resulted in a statistically significant pain reduction within 3 days, reduced opioid intake compared with placebo after hemorrhoidectomy [38].

In another randomized, multicenter, double-blinded study conducted by Smoot et al., to investigate the extent and time duration of analgesia effect achieved with lipid nanoparticle loaded bupivacaine in patients undergoing cosmetic and sub-muscular augmentation mammoplasty under general anesthesia, 136 patients were randomized to either a single dose of lipid nanoparticles bupivacaine 600 mg or bupivacaine HCl (0.5 % bupivacaine with epinephrine 1:200,000). Then, 1000 mg of acetaminophen was administered to all patients three times a day and rescue oxycodone as mandatory 3 days after surgery [39]. The primary end point and secondary outcome of this study are summarized in Table 4. Finally, this study reported that the cumulative amounts of opioid taken were significantly lower in the lipid nanoparticle bupivacaine group through 24 h and through 48 h as such the authors concluded that lipid nanoparticle bupivacaine is promising as regards efficiency compared with bupivacaine HCl and required less opioid intake [40].

Furthermore, another randomized, double-blind phase 3 clinical study on bunionectomy patients, and a total of 193 patients were randomized to receive either lipid nanoparticle bupivacaine 120 mg or placebo through wound infiltration. All the patients were sedated by administering propofol with Mayo block with up to 25 ml of 2 % lidocaine with epinephrine. The patients received either 180 mg (8 ml) of DepoFoam bupivacaine or the placebo, and 8 ml of 0.9 % sodium chloride, 30 min after the Mayo block was administered. The primary end point and secondary outcome of this study are summarized in(Table 4. Conclusively, the study showed that lipid nanoparticle bupivacaine provided improved pain relief and reduced intake of opioid after bunionectomy, compared with placebo [4].

Cohen et al. carried out an open-label cohort study to investigate the total opioid burden and health economic outcomes in adult patients undergoing open segmental colectomy [41] with anastomosis with general anesthesia who were administered with received lipid nanoparticle bupivacaine for postsurgical pain compared with those who were administered with patient-controlled analgesia (PCA) with opioids. The primary end point and secondary outcome of this study are summarized in Table 4. The results showed that the lipid nanoparticle bupivacaine group had significantly reduced total postsurgical opioid use than the PCA group [42].

A new product, EXPAREL™ (Pacira Pharmaceuticals Inc., NJ, USA), made up of DepoFoam (MLV) liposomal bupivacaine has been developed and according to a new clinical study, patients that were administered DepoFoam bupivacaine had decreased rescue analgesia urgency compared with plain 0.5 % bupivacaine at 24 h [43]. However, the calculated mean cumulative pain scores at rest or without rest were the same, as were rescue medication necessities at all other time points up to 3 days but it should be noted that muscular pain was reported in 6 out of 66 (9 %) patients administered with DepoFoam bupivacaine, but none was reported with plain bupivacaine [44].

Recent research works have demonstrated the efficacy of nanoparticle in a rat model where local analgesia was reported administered to nine of ten animals treated with bupivacaine-loaded nanofibers, with the effect of the LA been observed at day 1 and maximum effect observed at day 3; however, the observed effect was lost between 7 and 9 days. In addition, Chen and other researchers developed a sandwich-structure electrospun fiber mat that is made up of two outer layers and an inner layer consisting of PLGA/collagen fibers and PLGA fibers respectively containing two antibiotics drugs and one anesthetic drug, namely gentamycin and vancomycin and lidocaine, respectively leading to promising repair of infected wounds in a rat model carried out in vivo. Weldon and his colleagues also developed an electrospun nanofiber that provided a promising sustained local analgesia using bupivacaine [45].

Furthermore, and recently, there has been a development of local anesthetics skin-delivery systems using lipid nanoparticles, and this technique has been channeled into commercial formulations such as lidocaine and prilocaine cream, leading to modified release rate of drugs, increased bio-adhesive abilities, and reduced toxicity, thereby improving therapeutic potency [46].

Previous research works have developed a safe and sustained analgesia effect for 48 h after administration of local anesthetic injection of 2 % lipid nanoparticle lignocaine [47]. In addition, it was reported that after an epidural injection of lipid nanoparticle bupivacaine, there was a twofold increase in the time duration of the analgesia effects [48]. Furthermore, research studies in the field of intraoral topical anesthesia reported that lipid nanoparticle-encapsulated 2 % ropivacaine gel was as potent as 20 % benzocaine gel in decreasing pain incurred during needle injection and activating soft-tissue anesthesia [49]. Recently, in a randomized single-blinded, placebo-controlled research study, lipid nanoparticle-encapsulated ropivacaine formulations (1 %, 2 %) was not effective in decreasing the pain of needle injection into the palatal mucosa. In another blinded cross-over study, two anesthesia drug effects were compared and evaluated in intraocular patients [17]. The injection discomfort was compared between 2 and 3 % lipid nanoparticle-encapsulated mepivacaine with 2 % mepivacaine with 1:100,000 adrenalines and 3 % mepivacaine. The encapsulation of mepivacaine was responsible for the increase in the time duration of anesthesia drug effects and decreased the discomfort or side effects caused by these vasoconstrictor-associated formulations [50].

## Conclusions

The development of local anesthetic drugs has become an alternative to decrease pain and anxiety linked to invasive surgeries. Anesthesia drug like DepoFoam bupivacaine is a promising and efficient new method to deliver bupivacaine and may serve as a potent instrument in postoperative pain control. A good comparison between plain bupivacaine and lipid nanoparticle bupivacaine has demonstrated to showcase many advantages when used in wound infiltration that includes opioid-sparing effect, prolonged analgesia, higher patient satisfaction, prompt discharge, and reduced hospital bills.

Presently, different strategies and techniques have been purposed for local anesthetic drug delivery that include mostly lipid nanoparticles and formulations of hydrogels leading to a fast and specific delivery and prolongation of the duration of anesthesia; however, with the promising future of lipid nanoparticles application in bio-medical fields, more multicenter clinical trials are needed to be carried out.
